# Surgery combined with adenoviral p53 gene therapy for treatment of non-small cell lung cancer: a phase II study

**DOI:** 10.18632/oncotarget.22333

**Published:** 2017-11-06

**Authors:** Bo Deng, Tianyu Sun, Bo Tang, Shaolin Tao, Poming Kang, Kai Qian, Bin Jiang, Kun Li, Kunkun Li, Jinghai Zhou, Ruwen Wang, Qunyou Tan

**Affiliations:** ^1^ Department of Thoracic Surgery, Institute of Surgery Research, Daping Hospital, Third Military Medical University, Chongqing 400042, China

**Keywords:** adenoviral p53 gene, non-small cell lung cancer, radical surgery, recurrence, metastasis

## Abstract

**Objective:**

To assess the efficacy of radical surgery combined with recombinant adenoviral human p53 (rAd-p53) gene therapy in treatment of resectable non-small cell lung cancer.

**Method:**

A total of 163 patients with resectable NSCLC meeting the inclusion criteria were randomly assigned to two groups: radical surgery alone (S) and radical surgery plus surgical wound surface injection of 2 x 10^12^ rAd-p53 units (SP). All patients were followed up for at least 3 years for efficacy and safety. Study endpoints were loco-regional recurrence or distant metastasis (Rec-Met) rate as primary endpoints, and progression free survival (PFS), overall survival (OS) and safety assessments as secondary endpoints.

**Results:**

Recurrence or metastasis (Rec/Met) after surgery were 24/82 (29.27%) in SP group and 37/81 (45.68%) in S group. The difference in the Rec/Met rate was statistically significant (p = 0.0304) by chi-square test. The hazard ratios after adjusting of age and disease stage (S vs. SP) of PFS and OS are 1.772 (95% CI, 1.102 to 2.848) and 2.047 (95% CI, 1.109 to 3.377), respectively. The 3 years PFS and OS for SP vs. S were 71.9% vs. 46.9%, and 88.4% vs. 67.0%, respectively. Differences in PFS and OS between two treatment groups were significant with the p values of 0.0165 and 0.0191, respectively, using Log-Rank test.

**Conclusions:**

The wound surface injection of rAd-p53 showed efficacious effects in preventing recurrence or metastasis and improving PFS and OS after a radical surgery in patients with NSCLC.

## INTRODUCTION

In the past 30 years, the incidence and mortality of lung cancer significantly increased. Lung cancer is the leading cause of death for all kinds of cancer in men and women worldwide. The American Cancer Society’s estimates for lung cancer in the United States for 2016 are: about 224,390 new cases of lung cancer (117,920 in men and 106,470 in women) and about158,080 deaths from lung cancer (85,920 in men and 72,160 in women). In China, over 1.6 million of the new cases are diagnosed each year, accounting for 12.7% of new cases for all cancer types, and over 1.37 million of the lung cancer patients die each year. The non-small cell lung cancer (NSCLC) is the most common type of lung cancer and accounts for about 75% - 85% of all cases.

Radical surgery still remains the main and potentially curative treatment for the early stage NSCLC. The five-year survival rate is from 30% to 70% after a radical surgery depending on the disease stage [1–3]. Local recurrence or remote metastasis is the main cause of death after a radical surgery. It is estimated 30% to 80% of patients with NSCLC will develop local recurrence or remote metastasis in 5 years after surgery [4, 5]. Therefore, there is a need to further improve the NSCLC patients’ survival by preventing or delaying the recurrence or metastasis after radical surgery.

P53 gene is one of the most important tumor suppressing genes. It has multiple anti-tumor functions including blocking of the cell cycle, inducing cell apoptosis, inhibiting tumor angiogenesis and sensitizing tumor to chemo- and radio-therapy [6–8]. P53 mutations are common in NSCLC with occurrence of 70% [9]. Loss of p53 normal functions is associated with tumorigenesis, and chemo- and radio-therapy resistance [10]. Gendicine^®^, an antitumor gene product approved for marketing by the Chinese Food and Drug Administration (CFDA) in 2004, is a recombinant adenoviral p53 gene (rAd-p53), in which a wild type p53 CDNA was inserted in a replication-defective adenovirus type 5. A phase II study indicated that intratumoral injection of rAd-p53 can improve 5-years overall survival (OS) and progress free survival (PFS) when combined with radiotherapy in treatment of patients with advanced head and neck cancer [11]. It also has been shown that injection of rAd-p53 to the surgical wound could prevent or delay the recurrence of oral cancer after radical surgery [12]. Common adverse events associated with using rAd-p53 are self-limiting mild to moderate fever [11, 12]. So far, no serious adverse effect related to rAd-p53 has been reported.

The objectives of this study are to investigate the effects of surgical wound surface injection of rAd-p53 to prevent recurrence or metastasis after radical surgery for patients with NSCLC.

## RESULTS

### Patients and disease characteristics

From March 10 of 2012 to June 30 of 2013, a total of 180 of patients recruited, and 163 of the patients met the inclusion criteria and were randomly assigned to either one of the two treatment groups: 82 patients in the SP group and 81 in the S group. Patients’ demographics and baseline disease characteristic were summarized in Table 1 and Table 2 , respectively.

**Table 1:** Demographics

	Surgery plus rAd-p53 N=82	Surgery alone N=81
Age (years)		
Mean (SD)	58.4 (12.9)	57.1 (11.3)
Median (Min, Max)	61 (39-75)	59 (41-73)
Sex		
Male	57 (65.5%)	54 (66.7%)
Female	25 (34.5%)	27 (33.3%)
Smoking History		
Yes	48 (58.5%)	45 (55.6%)
No	34 (41.5%)	36 (44.4%)

**Table 2:** Disease Characteristics at Baseline

	Surgery plus rAd-p53 N=82 n (%)	Surgery alone N=81 n (%)
Histology Type		
Adenocarcinoma	37 (45.1)	32 (39.5)
Squamous	32 (39.0)	25 (30.9)
Large Cell	5 (6.1)	8 (9.9)
Undifferentiated	1 (1.2)	2 (2.5)
Other	7 (8.5)	14 (17.3)
Stage		
I	33 (40.2)	36 (44.4)
II	37 (45.1)	34 (42.0)
IIIa	12 (14.6%)	11 (13.6)
TNM stage		
Ia (T1N0)	15 (18.3)	14 (17.3)
Ib (T2N0)	18 (22.0)	22 (27.2)
IIa (T1N1)	19 (23.2)	17 (21.0)
IIb (T2N1, T3N0)	18 (22.0)	17 (21.0)
IIIa	12 (14.6)	11 (13.6)
ECOG		
0	47 (57.3)	48 (59.3)
1	35 (42.7)	33 (40.7)

### Surgery

For all the patients, the visible tumors were successfully removed by one of the surgical procedures: lobectomy, segmentectomy, or pneumonectomy. The detailed summary of the surgical procedures are showed in Table 3.

**Table 3:** Surgical Procedures

Surgery	Surgery plus rAd-p53 N=82 n (%)	Surgery alone N=81 n (%)
Lobectomy	63 (76.8)	58 (71.6)
Segmentectomy	11 (13.4)	10 (12.3)
Pneumonectomy	8 (9.8)	13 (16.1)

### Loco-regional recurrence and distant metastasis

The loco-regional recurrences (Rec) are the recurrent tumors after surgery, which located within the same hemi-thorax as the original tumor including ipsilateral lung, lymph nodes, the bronchial stump, and the pleura and chest wall. The recurrent tumors located remotely from the local region are considered as distant metastases (Met). At 3 years after surgery, there were 24/82 (29.27%) patients in SP group developing either Rec or Met (Rec/Met), compared with 37/81 (45.68%) patients in S group. There were 3 patients who had both Rec and Met in SP group and 8 patients who had both Rec and Met in S group, which could explain that the number of Rec and Met in SP group was 24 instead of 27 and the number of Rec and Met in S group was 37 instead of 45. The difference in Rec/Met rate is statistically significant (*p* = 0.0304) using chi-square test. The Rec/Met data was summarized in Table 4.

**Table 4:** Loco-regional Recurrence and Distant Metastasis

Rec/Met	Surgery plus rAd-p53 N=82 n (%)	Surgery alone N=81 n (%)
Any Rec	9 (11.0)	20 (24.7)
Lung Around Surgical Margin	5 (6.1)	13 (16.0)
Lymph Nodes	6 (7.3)	9 (11.1)
Pleura	4 (4.9)	7 (8.6)
Chest wall	1 (1.2)	3 (3.7)
Any Met	18 (22.0)	25 (30.7)
Brain	8 (9.8)	11 (13.6)
Bone	6 (7.3)	9 (11.1)
Liver	5 (6.1)	8 (9.9)
Adrenal	3 (3.7)	5 (6.2)
Rec and Met	24 (29.3)	37 (45.7)

### PFS and OS

At the time of this analysis, the minimal follow-up time was 3 years and the median follow-up was 3.6 years with a range of 3 to 4.8 years. The number of patients with an outcome of death (17) or Rec/Met (12) was 29 for the SP group, compared with a total 42 of deaths (26) or (14) in the S group. The 3-years PFS and OS for SP vs. S are 71.9% vs. 46.9%, and 88.4% vs. 67.0%, respectively.

**Figure 1:**
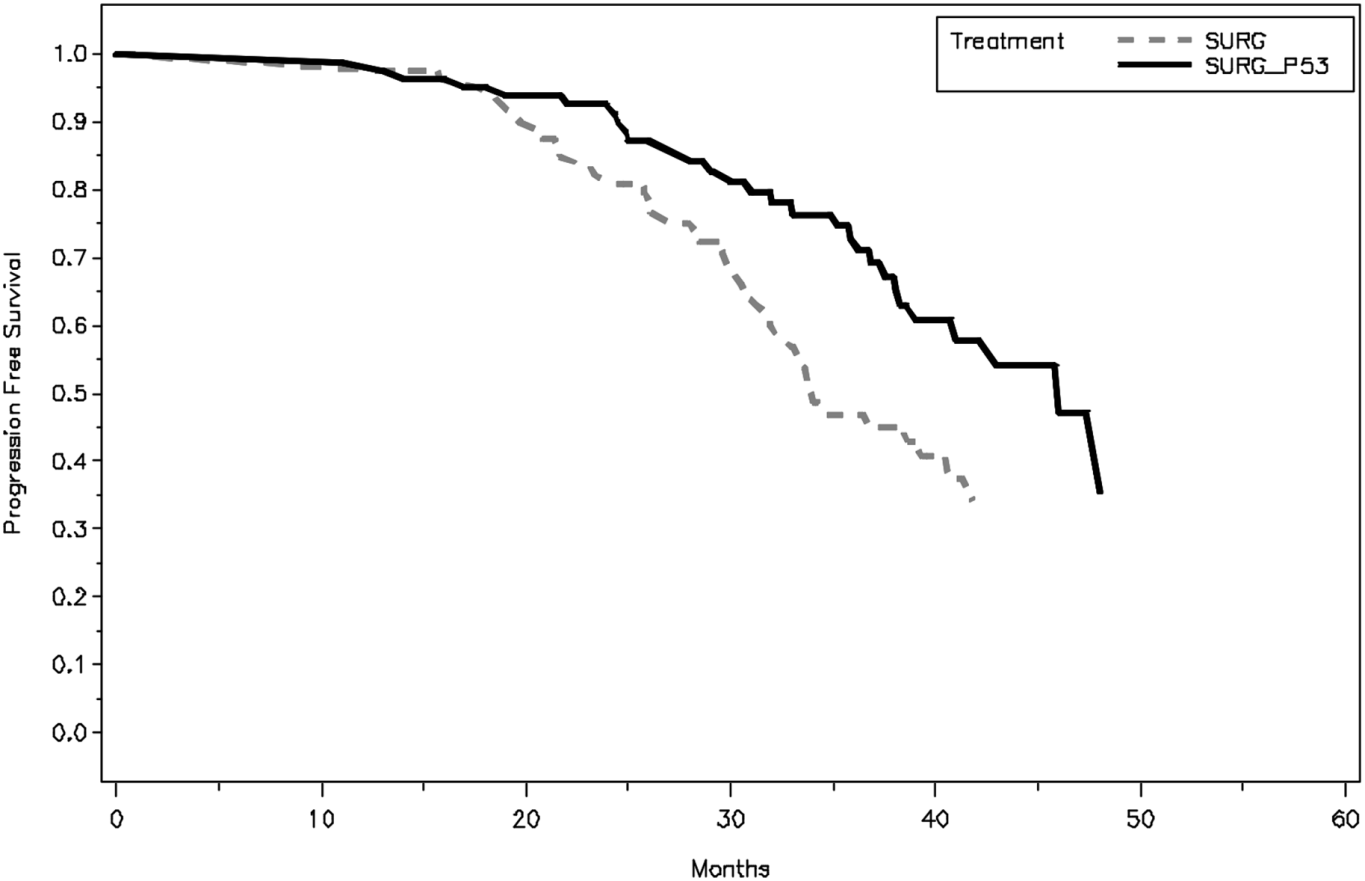
Kaplan–Meier plot of progression-free survival (PFS), assessed from surgery to the time when the last patient finished his 3-years follow-up

The difference in PFS between the two groups is statistically significant (P =0.0165) using log-rank test. The median PFS of SP group was 46 months with 95% CI of 38.2 months to 53.5 months, comparing to the S group with median PFS of 34 months with 95% CI of 31.8 to 41.9 months. The hazard ratio of S vs. SP group for PFS is 1.772 with 95% CI of 1.102 to 2.848 using Cox model adjusted by age and disease stage. The PFS plots are shown in the Figure 1.

**Figure 2:**
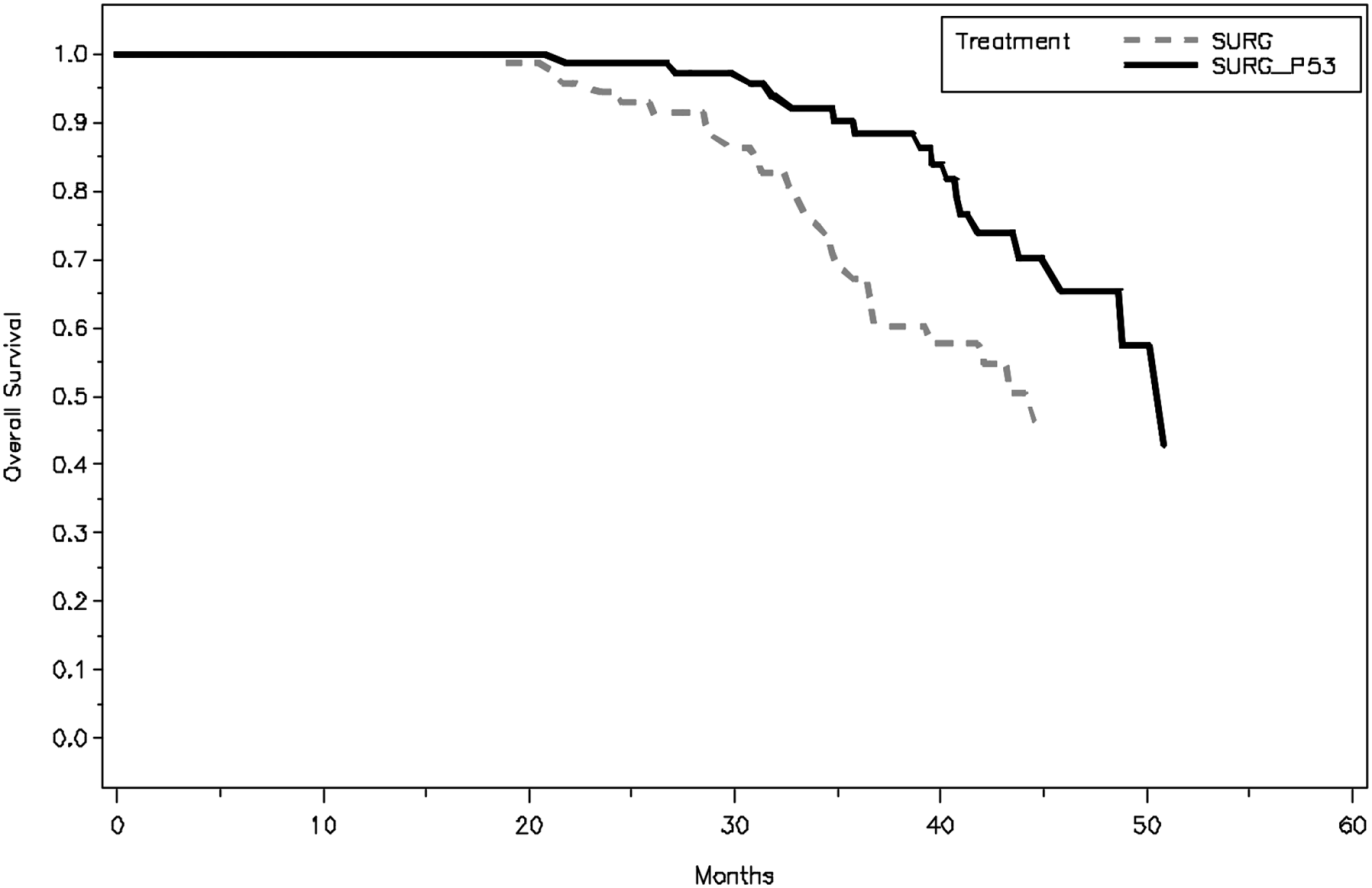
Kaplan–Meier plot of overall survival (OS), assessed from surgery to the time when the last patient finished his 3-years follow-up and his follow-up was the shortest

The difference in OS between the two groups is statistically significant (P =0.0191) using log-rank test. The median OS of SP group was 50.8 months with 95% CI of 45.8 months to 56.3 months, comparing to S group with median OS of 44.6 months with 95% CI of 36.8 months to 51.7 months. The hazard ratio of S vs. SP group is 2.047 with 95% CI of 1.109 to 3.377 adjusted by age and disease stage. The OS plots are shown in the Figure 2.

### Adverse events and complications

The adverse events and surgical complications were summarized in Table 5. Among them, only fever was classed as the rAd-p53 related adverse event.

**Table 5:** Adverse Events and Complications

	Surgery plus rAd-p53 N=82 n (%)	Surgery alone N=81 n (%)
Pneumonia	29 (35.4)	22 (27.2)
Respiratory failure	13 (15.9)	8 (9.8)
Hemorrhage	0	2 (2.5)
Bronchial fistula	0	1 (1.2)
Arrhythmia	5 (6.1)	4 (4.9)
Recurrent laryngeal nerve injury	1 (1.2)	0
Fever (>38°C)	49 (59.8)	6 (7.4)

## DISCUSSION

Local recurrence and remote metastasis are still the main cause of death after a radical surgery for NSCLC. Decrease in the Rec/Met rates will further improve the patients’ survival. One may immediately think of the method to reduce the rate was to choose an appropriate surgical procedure and completely remove the residual tumor cells according to accurate assessment of the extent of tumor invasion based on imaging scans and observation during surgery. But this method is mostly dependent on a surgeon’s experience and also tumor size and location.

A number of studies indicated that both pre- and post-surgery neoadjuvant and adjuvant can improve patients’ PFS and OS [13–16]. One randomized phase III study compared between surgery alone and surgery plus pre-operative cisplatin and gemcitabine for treatment of stage IB to IIIA NSCLC [17]. The hazard ratios for PFS and overall survival were 0.70 (95% CI, 0.50 to 0.97) and 0.63 (95% CI, 0.43 to 0.92), respectively, both in favor of chemotherapy plus surgery. In this report the authors reviewed several similar studies and all these studies showed pre-operative chemotherapy could improve the NSCLC patients’ survival. Suehisa H and Toyooka S reviewed the post-surgery adjuvant chemotherapy for completely resected non-small-cell lung cancer [18]. In this review, several studies showed the post-surgery adjuvant chemotherapy improved the 5-year survival from 4.1% to 11%. In this study, neither pre- nor post-operative chemotherapy was used because of the concern that serious adverse events from pre-operative chemotherapy might delay surgery, and that post-operative chemotherapy might be ineffective and could cause serious adverse effects. However, a long term follow up is needed to fully define the beneficial effects of rAd-p53.

Considering the multiple anti-tumor functions of p53 gene and the favorable safety profile of rAd-p53 we used rAd-p53 as an adjuvant therapy for completely resected NSCLC. The results showed that rAd-p53 significantly lowered the Rec/Met rate (*p* = 0.0304) and improved both PFS and OS.

The rAd-p53 related adverse event was self-limited fever. The Fever >38°C was observed in 59.8% of the SP group versus 7.4% of S group. The fever was from 38°C to 40.6°C, occurred 2 to 6 days after the surgery and usually lasted 2-5 days. To most of the patients, physical methods were effective in relieving the fever. No other rAd-p53 related adverse event was observed.

In conclusion, the wound surface injection of rAd-p53 showed efficacious effects of preventing recurrence or metastasis and improving both PFS and OS after a radical surgery in patients with NSCLC, and a better safety profile. These patients are still followed up to investigate the long-term beneficial effects of rAd-p53.

## MATERIALS AND METHODS

### Study designs

This was a phase II, randomized and open-labelled study to investigate the beneficial effect of the surgical wound surface injection of rAd-p53 after a radical surgery for patients with resectable NSCLC. The study was approved by the Ethics Committee of Daping Hospital of Third Military Medical University and registered in www.ClinicalTrials.gov (NCT01574729).

### Eligibility

All patients were histopathologically diagnosed with resectable stage I to IIA NSCLC, had Eastern Cooperative Oncology Group (ECOG) performance status of 0 or 1, were with normal hemogram, blood coagulation, liver and kidney function tests, and were over 18 years old. In addition, there were no surgery contraindications such as severe cardiac disease, uncontrolled diabetes mellitus. All the patients provided written informed consent.

### Treatment

Patients were randomly assigned to receive either a radical surgery alone (S) or a radical surgery plus surgical wound surface injection of rAd-p53 (SP). In order to completely remove the primary tumor lesions, a segmentectomy, lobectomy, or pneumonectomy and systematic mediastinal lymphadenectomy were performed according to the lesion size, location, and number of lesions. For SP group, after removing the primary tumor and performing complete hemostasis, 2 x 10^12^ viral units of rAd-p53 diluted into 10 - 15 ml of physiological saline containing 1:5000 epinephrine were evenly injected into the surgical wound surface by multiple injection points and multiple directions at one point. To avoid injection the medication into blood vessel, before injecting, pull back slightly on plunger to check for blood in syringe.

All participating patients were followed up for at least 3 years. In the first year after surgery, a chest x-ray and computed tomography scan were performed every 3 months, every 4 months in the second year and then every 6 months after 2 years.

### Statistical analysis

SAS 9.3 was used for analysis of data. The recurrence or metastasis (Rec/Met) rate was calculated and comparison by chi-square test was performed between the two treatment arms. The PFS was defined as the time from surgery to Rec/Met, or death, and OS was defined as from surgery to death. The PFS and OS were estimated using Kaplan–Meier methods and compared between the two groups using Log-rank test. The hazard ratio of PFS and OS (SP vs. S group) were estimated by Cox model with tumor stage and age as a covariate. All the safety data were summarized.
